# Methicillin-Resistant *Staphylococcus aureus* at Canoe Camp

**DOI:** 10.3201/eid1211.060363

**Published:** 2006-11

**Authors:** Kathryn Como-Sabetti, Kathleen Harriman, Billie Juni, Amy Westbrook, Elizabeth Cebelinski, David Boxrud, Ruth Lynfield

**Affiliations:** * Minnesota Department of Health, Saint Paul, Minnesota, USA

**Keywords:** Community-associated methicillin-resistant Staphylococcus aureus, CA-MRSA, antibiotic resistance, dispatch

## Abstract

We investigated a cluster of community-associated methicillin-resistant *Staphylococcus aureus* infections among persons at a wilderness canoe camp. Isolates from the investigation had identical profiles for susceptibility, pulsed-field gel electrophoresis, and toxins. Participants in activities that involve skin injury, person-to-person contact, and inadequate hygiene are at increased risk for methicillin-resistant *S. aureus* infections.

In 2000, the Minnesota Department of Health (MDH) initiated prospective active surveillance at 12 sentinel hospitals for community-associated methicillin-resistant Staphylococcus aureus (CA-MRSA) infections. Cases are classified as CA-MRSA if the patient lacks traditional healthcare-associated risk factors (hospitalization, surgery, dialysis, residence in a long-term care facility during the year before culture, or having an indwelling device at the time of culture). Although >80% of CA-MRSA infections involve skin or soft tissue, invasive disease that involves bones, joints, and sepsis can occur (*1**,**2*). During 2000–2004, 2% of CA-MRSA cases reported to MDH were joint infections.

Outbreaks of CA-MRSA have been associated with sports that require physical contact and result in frequent damage to skin (*3**,**4*) and with crowded settings (e.g., correctional facilities, military settings), where access to hygiene measures is limited (*5**,**6*). In response to a case cluster of CA-MRSA infections at a wilderness canoe camp, MDH performed an investigation to identify additional cases; MRSA colonization among staff, campers, and household members; and risk factors for infection.

## The Study

In August 2004, MDH was notified of 2 previously healthy 15-year-old male patients who were hospitalized for septic arthritis caused by MRSA; 1 had required treatment in an intensive care unit. The patients had been in the same group that participated in a 21-day wilderness canoe trip in northern Minnesota, USA. A third case-patient, a previously healthy 17-year-old female camper who had participated in an earlier 21-day trip and had had a skin infection without complications, was identified during the investigation (Table).

**Table:** Methicillin-resistant Staphylococcus aureus infections, canoe camp, Minnesota, USA, 2004*

Case-patient	Clinical progression
1	Reported mosquito bite on knee; evacuated from camp because of knee injury.
	Airlifted from clinic to hospital because of septic shock; required supportive care and knee debridement.
	Empirically treated with intravenous clindamycin.
	Had MRSA-positive joint culture and surgical specimens from knee, negative blood cultures.
	Treated with clindamycin, nafcillin, vancomycin, and linezolid.
	Hospitalized 11 d; had no long-term sequelae.
2	Knee stiffness and fever developed on the way home from camp.
	Did not report specific knee abrasion or injury.
	Admitted to hospital and underwent knee debridement.
	Empirically treated with intravenous cefazolin.
	Had MRSA-positive joint cultures and surgical specimens from knee.
	Treated with clindamycin.
	Hospitalized for 4 d; had no long-term sequelae.
3	Developed a forearm abscess 1 mo after trip.
	Did not report specific skin abrasion or injury.
	Skin culture was positive for MRSA.
	Treated with oral clindamycin.
	Was not hospitalized; had no long-term sequelae.

*MRSA, methicillin-resistant *Staphylococcus aureus*.

A case was defined as a clinically relevant, culture-confirmed, MRSA infection that occurred between June 1 and September 30, 2004, in a staff member, camper, or member of a camper's household. Colonization was defined as MRSA identified from the nares of these persons in the absence of MRSA infection.

MDH and local public health staff investigated the base camp. During the 21-day canoeing trip, participants spent 1 day at base camp at the beginning and end of the trip; the remaining time was spent canoeing, portaging, and camping on the trail. Camping groups were composed of 5–6 campers and a guide. Campers were of the same sex and were 15–17 years old. Campers and guides shared a common tent and did all activities together, but each had his or her own sleeping bag and towels. Campers and guides did not have access to running water or soap outside of base camp; the information on the wilderness area permit discourages use of soap, including biodegradable soap, within 150 feet of water.

Campers who had participated in the same, concurrent, or preceding canoe trips with the same itinerary and their household members were interviewed regarding health history, skin infections and injury, hygiene, clothing, camping behavior, and contact with other campers. Swabs of anterior nares were obtained from consenting staff, campers, and members of campers' households and were submitted to the MDH Public Health Laboratory. Isolates were identified by standard methods. Susceptibility testing was conducted by broth microdilution (PML Microbiology, Wilsonville, OR, USA) and interpreted according to Clinical and Laboratory Standards Institute (formerly NCCLS) criteria. Pulsed-field gel electrophoresis (PFGE) was performed by digestion with the restriction enzyme SmaI (*7*). Patterns were compared visually and with Bionumeric software (Applied Maths, Kortrijk, Belgium) to reference strains of S. aureus (*8*). Sequences encoding toxin genes (TSST-1, Panton-Valentine leukocidin, sea, seb, sec, and sed) were detected by PCR (*9**,**10*). Univariate analysis was conducted with EpiInfo version 6.04 (Centers for Disease Control and Prevention, Atlanta, GA, USA) using a significance level of p<0.05.

Of 21 campers, 19 (90%) were interviewed. The 2 case-patients had occasionally shared the same canoe. All campers reported having had frequent skin injuries, including insect bites, cuts, burns, blisters, and abrasions, during their trips. Campers reported having worn shorts most of the time and frequently having had wet skin, clothing, and shoes. No differences were identified between campers with and without CA-MRSA or between camping groups with and without CA-MRSA in terms of health history, use of antimicrobial drugs during the past year, skin infections and injury, hygiene, clothing, or camping behavior. Interviews were also conducted with 55 camper household members, representing 19 households. Members of 2 households could not be reached, and no information was obtained on the number of persons in these households. Interview responses of household members of case-patients did not differ from those of household members of the other campers and staff. No case-patient or colonized person reported a history of MRSA infection or colonization. One camper who was not a case-patient reported having had a positive blood culture for CA-MRSA in the prior year; this camper had been hospitalized with an infection that began as cellulitis and resulted in septic shock and was found to be not colonized at the time of the investigation. Also, the camp director informed MDH that in the prior year, 2 counselors had been treated for apparent spider bites at a local clinic; however, cultures had not been obtained.

Nares swabs were obtained for case-patients 2 and 3 but not case-patient 1 because he was receiving nasal mupirocin treatment. None of the case-patients was colonized with MRSA. Nares swabs were also obtained for 62% of campers without MRSA infection; of these, 1 female camper who had participated in a preceding trip was found to be colonized with MRSA. Swabs obtained from the anterior nares of 16 camp guides and base camp staff members showed MRSA colonization in 1. This staff member had not gone on any camping trips with the case-patients. Of nares swabs from 40 household members (including all 8 household members of case-patients), none was positive for MRSA.

All 5 MRSA isolates (from the 3 case-patients and the 2 colonized persons) were susceptible to clindamycin, ciprofloxacin, erythromycin, gentamicin, linezolid, quinopristin/dalfopristin, rifampin, tetracycline, trimethoprim/sulfamethoxazole, and vancomycin and had an MIC for mupirocin of <4 μg/mL. The isolates were USA400 pulsed-field type and were indistinguishable by PFGE (*8*). The isolate from the camper who had had an MRSA infection in 2003 was also indistinguishable by PFGE. All 2004 isolates contained genes encoding Panton-Valentine leukocidin, sea, and sec (*10*).

To prevent further infections, camp staff were given information about S. aureus, MRSA, and infection-prevention measures. Campers were instructed to wear long pants when possible, protect skin from injury (e.g., use insect repellent), report all suspected skin infections promptly, keep skin as clean as possible, use alcohol-based hand sanitizers, and not share personal items like towels and clothing. Frequently touched surfaces were cleaned with a bleach solution, and life vests were assigned to individual campers and disinfected between campers.

## Conclusions

CA-MRSA infections are increasingly reported and can be severe. Despite multiple outbreaks attributed to USA300, only a few reported case clusters have been caused by USA400 (*11**,**12*). USA400 does contain virulence factors, and although most cases have been mild skin and soft tissue infections, some have been severe and fatal (*13*). Furthermore, although the colonization rate was low for staff, campers, and household members (2.7%), it was higher than CA-MRSA colonization rates for the general population (*14*), as would be expected for case-patient contacts. S. aureus (including CA-MRSA) spreads easily among people in crowded settings, particularly when adequate hygiene cannot be maintained, both of which occurred in this camping setting.

Although anecdotal, suspected, and confirmed cases during the prior year are consistent with ongoing transmission associated with the camp, that spider bites were reported is noteworthy because CA-MRSA infections are often misdiagnosed as spider bites (*15*). Injuries and breaks in skin should be closely monitored in settings where medical care is not readily available. Methods for skin protection and hygiene should be developed and implemented for populations in settings where CA-MRSA transmission and infection can be anticipated.

## Acknowledgments

We thank Linda Van Etta, Jane Gilbert-Howard, Joni Kristenson, Jessica Buck, Pamala Gahr, Jane Harper, Miriam Shapiro, Joanne Bartkuis, and Anita Glennen for help with data collection.
